# Parental acceptance of brain health programs for preschool children: a mixed-methods study exploring barriers, facilitators and future approaches

**DOI:** 10.3389/fpubh.2024.1383270

**Published:** 2024-05-23

**Authors:** Lily A. Montague, Susan Hespos, Erin Mackenzie, Joyce Siette

**Affiliations:** ^1^The MARCS Institute for Brain, Behaviour and Development, Western Sydney University, Westmead, NSW, Australia; ^2^School of Education, Western Sydney University, Kingswood, NSW, Australia

**Keywords:** brain health, preschool, parental acceptance, dementia risk, public health

## Abstract

**Background:**

Recent research proposes that as much as 40% of dementia risk is amendable. Promoting healthy lifestyle behaviors in early life through educational methods can cultivate habits that may decrease dementia risk in later life. This study explores parental acceptance of brain health programs tailored for preschool children, aiming to identify barriers and facilitators affecting parental and child engagement.

**Methods:**

Mixed-methods cross-sectional study. Urban and suburban parents (*N* = 187, *M*_age_ = 37.3 *SD* = 5.53, range = 29) of children aged three to five years across Australia. Parents participated in an online survey containing both open and closed questions exploring their personal views and opinions on brain health programs for their preschool children. Descriptive statistics, multiple linear regression analyses, and thematic analysis were used to explore sociodemographic factors associated with parental program acceptance.

**Results:**

Most participants accepted a brain health program with over 98% agreeing a program would be useful for their child(ren). Participants with younger aged children were more likely to exhibit acceptance of a program (*β* = −0.209, *p* = 0.007). Three main categories emerged: dual home and preschool environments, the need for engaging brain health programs that were hands-on and screen-free, and addressing key barriers such as time and financial constraints to support implementation.

**Conclusion:**

Participants valued educating their children for a healthy life and viewed brain health programs favorably. This study contributes to early childhood education discussions, offering guidance for future generations’ brain health and wellbeing.

## 1 Introduction

Dementia is one of the fastest growing health concerns in the world (1). At present, research suggests that up to 40% of dementia risk can be modified (2). Existing research has primarily focused on the preventable aspect of dementia, with a recent study indicating the effectiveness of education on lifestyle behaviors in reducing dementia risk across the lifespan (3). The rise of dementia prevalence highlights the importance of educating children about healthy behaviors to ensure brain health is maintained in later life. However, a significant research gap exists regarding brain health programs designed for children, specifically preschoolers, in identifying factors that influence parents’ acceptance and potential implementation of such programs.

Brain health programs aim to provide education on various aspects of cognitive health. These programs aim to promote healthy habits and behaviors, raising awareness of healthy practices and their influence on maintaining a healthy brain. Existing programs such as the Finnish Geriatric Intervention Study to Prevent Cognitive Impairment and Disability (FINGER) (4) and the Multidomain Alzheimer Preventative Trial (MAPT) (5) have been developed and targeted toward adults aged 40 and higher, with some efficacy. Additionally, dementia literacy campaigns have been implemented to raise awareness within older cohorts (6). However, to our knowledge, there are no existing brain health education programs targeting early years (e.g., before primary school) that address healthy habits to reduce dementia risk. Within early childhood years, existing literature on general health education has primarily focused on building empathy for individuals with dementia (7) and enhancing understanding of dementia (8, 9), rather than directly addressing dementia risk reduction in early childhood. The limited research in this area highlights the need for further investigation and the development of interventions aimed at promoting healthy lifestyle habits and cognitive resilience from a young age.

Ensuring a robust educational foundation from childhood through adulthood has been associated with a decreased risk of dementia (2). Cognitive abilities tend to improve with education until a plateau is reached in late adolescence, emphasizing the potential impact of early life education on reducing the risk of later cognitive decline (10, 11). Despite the critical role of education in cognitive health, existing research has primarily focused on raising dementia awareness among children without addressing the promotion of healthy behaviors (8). While prior dementia awareness programs have proven successful with adolescents and children (12, 13), it is imperative to explore whether educating preschool children about healthy habits and brain health can similarly yield positive outcomes.

The self-efficacy theory, positing that behavioral and psychological changes result from modifying an individual’s sense of personal mastery or efficacy, is likely to influence parental acceptability of such programs (14). This theory implies that parents’ confidence in comprehending and effectively engaging with program content significantly influences their acceptance and engagement levels. Conversely, parents who do not perceive the benefits of the program are less likely to endorse these behaviors. With this, parents are known to hold the greatest influence on their children’s experiences and the environments they interact with during early life (15). Subsequently, parents are seen as the key facilitators in the development and implementation of healthy behaviors. Research examining parental acceptance of general health programs for children has highlighted various factors influencing parental decisions. Barriers often encompass concerns about safety, time and resource constraints (16), financial limitations, doubts about effectiveness (17) and prior experiences of health care (18). Moreover, logistical challenges such as transportation issues and scheduling conflicts can impede parental participation (18, 19). Conversely, facilitators of parental acceptance include clear communication regarding program benefits, involvement of trusted healthcare professionals, convenient scheduling options, parental motivation (20, 21) and accessible locations (18). Tailoring programs to align with the specific needs and preferences of parents and children, offering educational resources, and providing incentives have also proven effective in enhancing parental acceptance and active engagement in health initiatives for children (21).

Studies on dementia risk reduction programs have highlighted several key barriers that impede parents’ willingness to embrace lifestyle changes (22, 23). Among these obstacles, similarly, time constraints emerged as a prevalent challenge, alongside the presence of other pre-existing health conditions (22). Skepticism surrounding dementia risk, often rooted in misconceptions or insufficient understanding, was also seen to be prevalent. Another barrier being people lacking awareness about dementia and its importance (22, 23). These barriers highlight the need to explore parental preferences, inclusive of their barriers and facilitators, to ensure a program will be accepted and implemented effectively.

The present study aimed to (i) explore parental acceptance of brain health programs designed for preschool aged children; (ii) understand the barriers and facilitators influencing parental and child engagement, and (iii) identify parental preferences on the main components of an educational program, such as preferred delivery format and setting.

## 2 Methods

### 2.1 Study design

The study utilized a 15-minute, online, cross-sectional survey containing both open and closed questions. The survey was approved by the Western Sydney University Human Research Ethics Committee (H15440).

### 2.2 Participants

Parents with at least one child aged between three and 5 years were recruited across all states in Australia to undertake the survey. Physical flyers were distributed to early learning centers, preschools, and playgroups across New South Wales, while electronic flyers and e-newsletters were distributed through the research team’s networks including Western Sydney University’s institutional networks and national parent groups on Facebook and established parent associations (e.g., Ryde District Mums and Northern Beaches Mums). Participants needed to meet eligibility criteria which included being over the age of 18 years old, parenting of a child(ren) aged three to five years old, communicating in English, and providing informed consent. An incentive was used to promote the completion of the survey, comprising of a $200 gift voucher provided at random to one participant at study completion. The study recruited 257 individuals, ranging from ages 25–54 years (*M*_age_ = 37.3, *SD* = 5.53), with 187 participants completing the survey (72% completion rate). Sample characteristics are summarized in Table 1. Parents were mostly aged between 35 to 44 years (65.4%), women (94.1%) and living in a major city (92%). Most had completed a Bachelor’s degree as their highest level of education (43.3%) and were born in an English-speaking country (75.4%). Almost half of the sample (48.7%) were from a high socioeconomic background. Most respondents were working (86.1%), with a majority (94.7%) being married or *de facto* relationships. Almost half (47.1%) of the participants reported having a relative or someone they know suffer from a neurological disease.

**Table 1:** Summary of participant sample characteristics (*N* = 187).

Variable	*N* (%)	*M* (*SD*)
Age (years)		
25–34	53 (28.3)	
35–44	122 (65.2)	
45–54	12 (6.4)	
Gender		
Male	9 (4.8)	
Female	176 (94.1)	
Prefer not to say	2 (1.1)	
Country of birth		
English-speaking country	141 (75.4)	
Non-English-speaking country	46 (24.6)	
Location		
Major city	172 (92)	
Regional area	15 (8)	
Socioeconomic status		
1 (lowest)	15 (8)	
2	22 (11.8)	
3	36 (19.3)	
4	201 (11.2)	
5 (highest)	93 (49.7)	
Highest level of education		
Non-graduate	38 (20.3)	
Graduate	148 (79.1)	
Prefer not to say	1 (0.5)	
Working Status		
Working	161 (86.1)	
Non-working	26 (13.9)	
Family structure		
Married/*De facto*	177 (94.7)	
Single/other	10 (5.3)	
Average age of children		4.28 (1.76)
Relative/someone with brain disease		
Yes	88 (47.1)	
No	97 (51.9)	
Prefer not to say	2 (1.1)	

### 2.3 Materials and procedure

The online survey was available from the 29th of June 2023 to the 21st of August 2023. The survey was created using the Qualtrics platform which included 19 closed-ended questions and nine open-ended questions. A forced response was applied to the closed-ended questions, providing an option for participants to skip over the open-ended questions to circumvent attrition. The survey is available in the Appendix.

**Demographics**: there were questions relating to age, gender, place of birth, postcode, highest level of education, family structure, and the number and age(s) of their children. Additional questions were asked regarding their child(ren’s) current lifestyle habits, such as their sleep hygiene and physical activity as well as their children’s current level of brain health education, for instance: at pre-school, home, or childcare. Socioeconomic status and locality were calculated using the index of relative socio-economic advantage and disadvantage (IRSAD) based on the participants’ suburb or postcode and were grouped in quantiles (24).

**Acceptability of brain health programs**: This group of questions was prefaced with an outline of what brain health programs are and what they aim to achieve. Questions assessed parents’ willingness to implement a brain health program alongside their perceived usefulness of the programs using a multiple-choice format with a yes or no option. An example question that was asked “Do you see brain health programs as being useful for your children?.” An open-ended response format followed to seek parents’ explanation if they selected ‘no’ to the above question.

**Delivery and modality preferences**: This section explored parents’ views and opinions on the location and format of a potential brain health program. Questions assessed what setting was most preferred by parents (home, preschool, or both). Preferred formats of a program were then explored by providing a list of five potential modalities and asking parents to rank modalities in order of preference. Open-ended questions were asked in this block requesting parents to provide explanations for their choices, while also offering an option to suggest further modalities to be considered for a program. An example open question was “Why do you prefer to have a program held in this space?”

**Barriers and facilitators**: A 6-point Likert scale derived from Hesketh et al. (15) was used to assess parents’ perceptions of barriers to accepting and implementing programs. Parents were asked to select their level of agreement with each statement (1 = strongly agree to 6 = disagree) for example, “I want an easily accessible brain health program” and “I do not want to spend too much money on brain health programs.” This part also explored participants’ perceived facilitators in the form of a multiple-choice question asking whether they had drivers or motivators to implement healthy lifestyle changes in their home with their children. Dependent on the answer (yes/no), an open-ended question followed up asking parents to elaborate, for instance, “What makes you passionate about healthy lifestyle habits for your child(ren)?” or “What makes you unmotivated about healthy lifestyle habits for your child(ren)?.” This block concluded with an option for parents to share any additional open-ended comments.

### 2.4 Analytical design

#### 2.4.1 Quantitative analysis

Descriptive statistics included the mean, standard deviation, frequency, and percentage of responses for demographics, acceptability/usefulness, preferred format of modality, setting delivery preferences of programs, and barriers/facilitators. A multiple linear regression analysis was employed to investigate the sociodemographic factors that influence the acceptability of brain health programs. The regression model was adjusted for various covariates, including country of birth, total barriers, gender, socioeconomic status, highest level of education, and the average age of children. Assumption testing was carried out prior to interpreting the results of the regression. The assumption of independence of observations was met by the design of the study as all parents were unrelated due to being surveyed across the nation. The assumption of multicollinearity was met as all tolerance values were above 0.2 (25). Singularity was absent as there were no perfect correlations between the independent variables (26). There were nine possible multivariate outliers as their Mahalanobis distance exceeded 22.46 which is the critical Chi-squared value for six predictors (*α* = 0.001), however, none of these cases were seen to exert undue influence. The standardized residuals for these cases were within the +/− 3.29 range (25). The assumption of normality of residuals was met as the histograms and Q–Q plots demonstrated normal distribution (25). To assess the assumptions of linearity and homoscedasticity of residuals, the scatter plot of standardized residual values versus standardized predicted values was visually inspected. The standardized residuals were evenly distributed from low to high values of predictor and non-linear pattern was not observed, thus the assumptions were met (25). The multiple linear regression analysis was performed using SPSS (58) and the significance level was set at *p* < 0.05.

#### 2.4.2 Qualitative analysis

Thematic analysis was used to analyze the qualitative data obtained through the nine open-ended questions. Factors shaping the acceptability of brain health programs were analyzed using Braun and Clarke’s (27) 6-step approach. This involved multiple stages, including data familiarization, generating initial codes, searching for themes, reviewing themes, defining/naming themes, and thematic report production. Two researchers (LAM and JS) initially coded the data and compared their findings for similarities and disagreements. There were no disagreements in the analysis, however, if any arose, a third researcher (EM) would have resolved it. Responses to all open-ended questions were coded, once codes were established themes were generated by grouping similar questions into the one theme. This was carried out to ensure the themes were comprehensively addressed.

Responses were first coded as (1) home; (2) preschool; (3) both. One first-order theme was developed: Parental Setting Preference. Comments that stated their location preference were further coded under the following second-order subthemes: Trust in Professionals, Reinforcement, Involvement, Engagement, and Controlled Environment. Responses were assigned to as many subthemes as were appropriate to cover content, for example, the comment “Preschool so he can learn with peers and home so that I have the knowledge to help him further develop this skill” was coded under the theme ‘Parental Setting Preference’ and the subthemes ‘reinforcement’ and ‘involvement’. Responses were analyzed using a purpose-designed Microsoft Excel V16.51 spreadsheet.

## 3 Results

### 3.1 Sample characteristics

Over half the sample reported their child(ren) had already been exposed to brain health information (56.1%), of which preschool/school education was the most common source for obtaining brain health information (47.1%) (Table 2). The majority (83.4%) of participants reported being accepting of brain health programs, although almost all (98.4%) believed a program would be useful for their child(ren). Most participants preferred education to be undertaken both at pre-school and at home (47.7%), with a fifth (22.5%) favoring a program delivered exclusively at preschool.

**Table 2:** Summary of parental acceptability, motivation, setting preferences, and children’s current brain health education.

Variable	*N* (%)
Exposure to brain health education	
Yes	105 (56.1)
No	82 (43.9)
Source of exposure	
Preschool/school	88 (47.1)
Children’s book	77 (41.2)
Educational apps/games	48 (25.7)
Online resources	32 (17.1)
Pediatrician/healthcare provider	28 (15)
Community health programs	20 (10.7)
Public health campaigns	13 (17)
Other	8 (4.3)
Acceptability	
Yes	156 (83.4)
Maybe	20 (10.7)
No	11 (5.9)
Usefulness	
Yes	184 (98.4)
No	3 (1.6)
Setting preference	
Both	133 (71.1)
Pre-school	42 (22.5)
Home	7 (3.7)
Present drivers/motivators	
Yes	154 (82.4)
No	33 (17.6)

Table 3 displays a summary of children’s existing habits as reported by participants. The most common lifestyle habits as reported by participants were socializing with peers (88.8%), physical activity (84.5%), mindfulness/stress management (17.1%) and education on the importance of healthy behaviors (19.3%).

**Table 3:** Summary of children’s existing healthy habits.

Variable	Yes *N* (%)	Somewhat *N* (%)	No *N* (%)
Sleep hygiene	121 (64.7)	63 (33.7)	3 (1.6)
Physical activity	158 (84.5)	29 (15.5)	0 (0)
Healthy eating	103 (55.1)	79 (42.2)	5 (2.7)
Education on healthy behavior importance	36 (19.3)	93 (49.7)	58 (31)
Mindfulness/stress management	32 (17.1)	99 (52.9)	56 (29.9)
Socializing with peers	166 (88.8)	20 (10.7)	1 (0.5)

**Figure 1:**
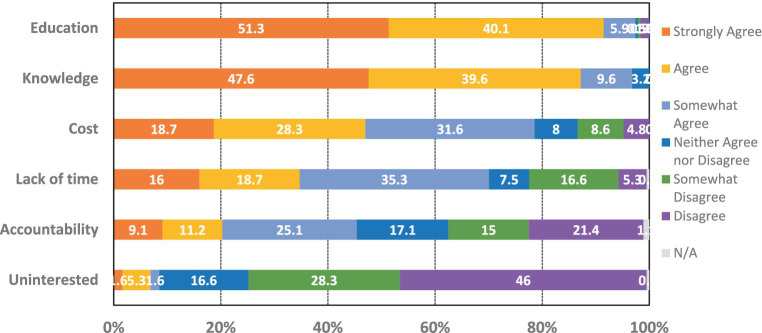
Barriers to implementing brain health program.

Figure 1 presents the participants’ perceived barriers to the implementation/adoption of brain health programs. The most prevalent barrier was the need for education (51.3%), followed by lack of knowledge (47.6%), and need for accessible programs (50.3%).

### 3.2 Multiple linear regression

A multiple linear regression was conducted to explore the factors influencing the acceptability of brain health programs with total barriers, gender, place of birth, socioeconomic status, level of education, and the average age of children as the predictor variables (Table 4) findings showed that the overall model was statistically significant, *F*(6,177) = 2.47, *p* = 0.026, explaining a small amount of variance, *R*^2^ = 0.077. Specifically, the average age of children was a significant predictor of acceptability, (*β* = −0.209, *t* = −2.75, *p* = 0.007), indicating that parents with younger aged children exhibited greater acceptance of brain health programs. The other predictors did not show significant associations with acceptability.

**Table 4:** Multiple linear regression analysis with acceptability as criterion variable.

Predictor	*SE*	*β*	*t*	*p*
Total barriers	0.03	−0.119	−1.63	0.105
Gender	0.19	−0.057	−0.756	0.450
Place of birth	0.09	−0.113	−1.54	0.125
Socioeconomic status	0.03	0.033	0.44	0.655
Level of education	0.10	0.039	0.62	0.615
Child average age	0.02	−0.209	−2.75	0.007

### 3.3 Thematic analysis

Several recurring themes emerged from the open-ended responses to the survey. These themes provided additional insights into parental preferences of setting and program modalities, while also providing awareness of their perceived barriers and facilitators to implementation. Three main themes were identified. ‘Parental setting preference’ encapsulated participants’ views on their optimal implementation setting. ‘Need for engaging, non-screen-based brain health programs’ explored participants’ reasoning behind their preferences for the varied modalities and solicited their input on alternative modalities. ‘Need to address perceived barriers and facilitators’ captured parents’ perceived barriers to adoption while also examining the facilitators that promote adoption.

#### 3.3.1 Parental setting preference

One hundred and eighty-seven participants explained why they selected their preferred setting for the delivery of a program. Parents who preferred a program held solely at preschool described the benefits of receiving information from trusted professionals. Parents also described the importance of their children learning in an environment with their peers. For example, Participant 32 stated a program held at preschool meant the program will be “Professionally taught and children are keener to listen to important information at school settings with peers,” and Participant 78 suggesting “We can have professionals at preschool to conduct the program with peers so that it can be more engaging.” Alongside these perceived benefits, time constraints due to competing priorities were seen to be alleviated by the option to have a program held solely at preschool, with Participant 40 noting, “I do not have the time to undertake a program at home” and Participant 81 stating “… I do not have enough time or energy to do it at home.”

On the other hand, some parents preferred a program to be conducted solely at home. This selection was influenced by their ability to control the environment for their child, and valuing working through the information based on the needs of their child, Participant 2 stating “I can work through it with them.” Parents also appreciated the ability to facilitate a safe space with no distractions, allowing for questions to be addressed more promptly. This was displayed through Participant 3 highlighting “Quiet and no distractions” and by Participant 4, “Able to have more child-led discussions and answer as many questions as needed.”

The combination of the two locations, home, and preschool, proved to be the most preferred option, with parents highlighting its mutual benefits in strengthening information, offering consistency and reinforcement, and fostering deeper consolidation. This was highlighted by Participant 11, “Preschool because of confidence in their education, at home as we can learn how to help with this and introduce practice to everyday life.” Additionally, parents also identified that the home-based component of a program would enable them to be involved in what the children are learning about, and to provide further consolidation. For example, Participant 41 stated “She is at school a lot so it’s a great time to use it [a brain health program], then to follow it up with home programs would solidify learning and engage parents.”

#### 3.3.2 Need for engaging, non-screen-based brain health programs

One hundred and eighty participants offered explanations for their preferred program modality while suggesting additional options. A few parents opted for an app/electronic based program, with Participant 9 noting, “My son … loves his iPad so I feel an app would work best.” However, a quarter of (25%) preferred programs that avoided electronic device usage due to an inclination for decreased screen time. These parents viewed programs delivered via devices as being ineffective in helping children to consolidate new information: “Any screen-based learning would be my least preferred option … learning simply does not stick …” (Participant 33), and “Probably do not need more screen time and to help message sink in” (Participant 31).

Due to this, parents highlighted the necessity and partiality for hands-on learning as expressed by Participant 43. “Prefer activities the child is participating hands-on in for effectiveness” through such as sensory activities and arts-based learning. Others highlighted the practicality of visual and storytelling activities for specific age groups: “Kids at this age tend to be more visual ….” (Participant 178), and “Hands on experience would be more beneficial for this age group with a story to introduce and a film to back it up with” (Participant 48). Similarly, it was common for the ranking of items to be based on what they thought their child(ren) would be most receptive to, with Participant 14 stating, “My children love sensory activities,” Participant 24 commenting, “My kid likes books,” and Participant 57 noting, “… kids at this stage loves to draw and be creative.” Parental preferences indicate the need to tailor strengths-based brain health programs to align with the individual interests and preferences of each child.

Different modalities of a brain health program were also suggested by parents. Parents recognized the importance of varied and engaging methodologies to promote children’s brain development, with modalities that incorporate role play, music, and play being highly regarded, for instance, Participant 51 suggested, “Something play based, or drama based where the children are actively involved in role play …,” with Participant 52 further suggesting, “Songs to sing at the points of brain health behaviors, like at sleep or eating,” and Participant 50, “Tactile learning activity, e.g., simple card tasks like matching, go fish.” This further emphasized the need for customized and adaptive programs, recognizing that each child varies in their receptivity and what aligns best with the family’s preferences.

#### 3.3.3 Need to address perceived barriers and facilitators

One hundred and sixty-six parents highlighted critical perceived barriers to promote their children’s engagement in brain health initiatives. These factors suggest the multifaceted challenges parents face and provided new insights into the complexities of integrating brain health initiatives into daily routines. The issue of time emerged as the most significant barrier, “Time is the biggest barrier, especially with busy working lifestyles and routines …” (Participant 43). Many parents, especially those with work commitments and demanding routines articulated their struggles in finding dedicated time for brain health activities. As noted by Participant 35, “… Getting home at 6 pm then the dinner, bath and bedtime routine can be hectic …,” demonstrating the challenges of integrating a brain health program into existing family routines.

Parents also expressed concerns about the perceived complexity of implementing brain health programs at home. Factors such as unexpected sickness, dealing with tenacious children, and children arriving home tired were identified as key obstacles, participants described this as “I guess anything unplanned like sickness” (Participant 96), “Child’s personality trait especially stubborn-ness!” (Participant 52), and “Children … come home tired and may not have the brain capacity to indulge in further stimulating activities” (Participant 43).

Additional responses further identified financial constraints and a lack of in-home support as common barriers to implementing brain health programs. Parents mentioned financial challenges and the need for external assistance within the home environment to facilitate implementation, as highlighted by Participant 9, “Money problems” and Participant 74, “I do need help in the home.” Competing priorities were also a noted factor, “Other siblings schedule” (Participant 59) and “Managing the conflicting needs of three children …” (Participant 80). Furthermore, parents also highlighted a lack of knowledge or awareness about brain health particularly with the other parent, “While I can implement … the other parent is reluctant and dismissive” (Participant 37), and “Both parents not being onboard” (Participant 94).

## 4 Discussion

The present study aimed to explore acceptability of brain health programs in parents of children aged three to five years, specifically focusing on preferred setting and delivery mode, and perceived barriers and facilitators. Parents expressed acceptance of a prospective brain health program, even in the face of potential multifaceted challenges, such as time constraints, contextual environments, and children’s attitudes. Recognizing this resilience suggests that with careful consideration and targeted strategies, effective program implementation can navigate and mitigate these challenges, ultimately contributing to the promotion of preschool children’s brain health and well-being.

Brain health programs targeting preschoolers’ risk reduction behaviors are a new concept. While activities in such programs are consistent with global trends in promoting early childhood interventions for cognitive and physical well-being [e.g., physical activity, (28–30), mental health, (31, 32)], this is the first comprehensive effort to specifically address brain health in preschool-aged children. The high acceptance rate observed in this study aligns with findings from other international initiatives, such as the FINGER (33) and international strategies like Massive Open Online Courses (34) focusing on brain health. However, unlike online educational programs that often lack direct interaction and hands-on activities, the preschool brain health programs described here emphasize experiential learning and active engagement, addressing a key gap in existing digital interventions. Furthermore, the identification of barriers such as time and financial constraints echoes findings from similar studies globally, indicating the need for tailored strategies to overcome these obstacles and ensure program accessibility and sustainability.

Our study found that parents exhibited a high level of acceptance for brain health educational programs, indicating their agreed utility in instilling healthy habits in preschool-aged children. This positive reception can be attributed to the alignment of these programs with existing national public health policies, including initiatives like the Early Years Learning Framework (35), National Quality Standard (36), Munch and Move Program (37), Get Up & Grow resources (38), SunSmart Program (39), and Staying Healthy in Childcare guidelines (40). These governmental efforts reflect a broader commitment to promoting healthier lifestyles and well-being among preschool-aged children in Australia. By aligning with established guidelines and frameworks that prioritises early childhood health and development, these programs gain credibility and trust among parents (41). This alignment fosters a sense of legitimacy, enhancing parents’ confidence in the efficacy and safety of the programs (42, 43).

While parents identified implementation barriers, their overall acceptance and motivation to use the program remained positive. Notably, time constraints and knowledge barriers emerged as the primary hurdles to implementation, aligning with previous research (44). This displays the importance of educating parents about both program content and outcomes. The concept of program accessibility resonates with the tenets of adult learning theory (45), which postulates that adults are more likely to engage in educational activities that are convenient, flexible, and tailored to their individual needs. In the context of parents, whose schedules are often constrained by work and caregiving responsibilities (46), an accessible program that accommodates their existing routines can become a vital catalyst for implementing programs for their children. Furthermore, acknowledging parents’ constrained schedules, an accessible program tailored to their needs can serve as a crucial catalyst for effective implementation (47).

Our results identified the importance of educating parents on what to do with, and how to carry out a program. This approach aligns with the self-efficacy theory (14, 48) which posits that if parents perceive the brain health program as complex or difficult to implement, their self-efficacy regarding their ability to implement the program may decrease. This leads to lower acceptability of the program and decreased implementation, as they may doubt their capability to understand the knowledge effectively. Similarly, when parents perceive the program material as intricate or challenging to understand, their self-efficacy regarding their ability to grasp and apply this information may decrease (49). This, in turn, can lead to lower program acceptability and decreased implementation. This suggests that if the program is designed in a way that enhances parents’ confidence in their capacity to comprehend the content, their acceptability and the implementation of the program may increase. Thus, if parents feel confident in their ability to apply the brain health principles from the program in their daily lives with their children, they are more likely to accept and adopt the program. However, if they doubt their capability to incorporate the principles, their acceptability and implementation may decrease. This connection highlights the importance of ensuring parents feel confident in their comprehension of program content, and ensuring programs are designed in a way that compliments and flows seamlessly into their daily lives.

The observed correlation between children’s age and parental acceptance of brain health programs prompts a deeper exploration into potential underlying factors. One consideration is the notion of intensified parental investment in cognitive stimulation for younger children, particularly firstborns, as supported by Luo et al. (50) and the concept of the “firstborn advantage” articulated by Chutiyami et al. (51). This trend thus could be attributed to heightened parental enthusiasm and vigilance, driven by the desire to provide optimal developmental opportunities for their firstborns (52).

However, the lower levels of acceptance among parents with older children necessitate further examination. One plausible explanation might involve the evolving dynamics of parenting. Parents with older children may encounter challenges related to conflicting schedules, increased demands on their time, and perhaps a perceived reduction in the need for intensive cognitive stimulation as they grow more accustomed to parenting (53). Indeed, parental experience might lead them to believe that their children will develop regardless of specific interventions, or they may feel more confident in providing cognitive stimulation themselves without relying on formal programs. Furthermore, the accumulated experience and knowledge gained from raising older children may also contribute to a sense of confidence or perceived adequacy in providing cognitive stimulation without the need for formal programs (54). Additionally, parents with older children might have faced shifting priorities, such as increased involvement in school-related activities or extracurricular pursuits, limiting their openness to additional cognitive stimulation programs for the younger child (55, 56). Indeed, parents with older children may perceive such programs as less necessary or impactful compared to parents with younger children who may be more receptive to additional support and guidance. However, our study did not gather information regarding the years of parenting experience of the participants, which may go beyond how many years their preschool-aged child has if they have other children, but rather the average age of their children. Future brain health educational programs should therefore consider how child age and developmental stage, specific needs, and challenges parents face at different stages of their parenting journey contribute to the design and implementation of effective interventions, ultimately enhancing program outcomes.

### 4.1 Limitations

The present study has several limitations, including a relatively small sample size and an unequal distribution of gender (4.8% male respondents). Further research is encouraged to incorporate the views of additional stakeholders such as educators and children alongside more males, to enable broader perspectives and assist in identifying whether there are any further differences in parental acceptability. Despite the survey being non-identifiable, parents may have provided answers they perceived would be socially acceptable or expected answers by the researchers, thereby introducing the potential for social desirability bias and demand characteristics (57). While the use of a questionnaire limited the depth of information collected despite including open-ended questions, additional studies using supplementary interviews could be beneficial in gaining deeper insights.

### 4.2 Implications

This study is one of the first, to our knowledge, to explore the potential of a brain health program within a preschool cohort. Our findings highlight the readiness of parents to embrace brain health programs, with those having young children particularly motivated and inclined to prioritize early cognitive development. The high acceptability displayed by parents indicates their willingness to provide brain health education to their children, reinforcing the imperative for comprehensive brain health programs that use family-centered approaches to address parents’ multifaceted perceived challenges. Recognizing and addressing these challenges is essential to ensure the successful adoption and sustained impact of brain health programs among preschool aged children.

### 4.3 Future directions

When constructing a program in the future, our findings suggest that it is important to ensure it is accessible, affordable, and time considerate, with the ability to be engaging and held both at preschool and home interchangeably and solely. To ensure maximum success in adoption, we suggest that future programs incorporate hands-on learning and avoids screen time, and educates parents about the program and its expected outcomes. Additionally, building strong parental confidence in their ability to deliver and understand a program is essential for successful implementation.

## 5 Conclusion

The present study demonstrated that brain health programs, while holding a large potential to educate and introduce healthy lifestyle habits for preschool children, may be under supported by the lack of discussion pertaining to this cohort. Parents acknowledged a need to educate their children through promoting a healthy life trajectory and found brain health programs as a highly acceptable means of achieving this. Collectively, these findings highlight the importance of tailoring interventions that prioritize program accessibility, comprehensibility, and alignment with parents’ diverse lifestyles. By addressing these factors, policymakers and educators can design more effective and inclusive brain health programs that empower parents and foster their confidence in nurturing their children’s cognitive development. This research contributes to the broader discourse on early childhood education and offers initial guidance for enhancing the brain health, wellbeing, and developmental outcomes of future generations.

## Data availability statement

The original contributions presented in the study are publicly available. This data can be found here: https://doi.org/10.26183/n9vq-5r55.

## Ethics statement

The studies involving humans were approved by Western Sydney University Human Research Ethics Committee. The studies were conducted in accordance with the local legislation and institutional requirements. The participants provided their written informed consent to participate in this study.

## Author contributions

LM: Conceptualization, Data curation, Formal analysis, Investigation, Validation, Visualization, Writing – original draft, Writing – review & editing. SH: Supervision, Writing – review & editing. EM: Supervision, Writing – review & editing. JS: Conceptualization, Investigation, Project administration, Resources, Supervision, Validation, Writing – original draft, Writing – review & editing.
