# Supplementary material for: Parental acceptance of brain health programs for preschool children: a mixed-methods study exploring barriers, facilitators and future approaches
Source: Front Public Health. 2024 May 23;12:1383270. doi: 10.3389/fpubh.2024.1383270 (PMC11177877; doi:10.3389/fpubh.2024.1383270)
Supplement: Supplementary file 1 [file Data_Sheet_1.pdf]

![frontiers]

# *Supplementary Material*

## 1 Survey

| Question | Response |
|---|---|
| Brain Health Programs aim to educate preschool aged children (i.e., between 3 and 5 years) and their families about the importance of better brains. It focuses on how lifestyle habits can build stronger brains and encourages children to engage in brain healthy behaviours. Would you be willing to use a brain health program for your child? | Yes (1)<br>Maybe (4)<br>No (3) |
| Could you please explain why you are hesitant/unwilling to use a brain health program with your child? | OPEN ENDED |
| Do you see brain health programs as being useful for your children? | Yes (1)<br>No (2) |
| Could you please explain why you do not feel brain health programs can be useful? | OPEN ENDED |
| Where would you prefer a brain health program to be held? | Preschool (1)<br>Home (2)<br>Both (3)<br>Other (4) ------- |
| Why do you prefer to have the program held in this space? | OPEN ENDED |
| Brain health programs can be delivered in different formats. Please rank by dragging the below formats in your order of preference | ______ Story Book (1)<br>______ Electronic Application (2)<br>______ Short Animation Film (3)<br>______ Sensory Activity (4)<br>______ Arts Based Delivery (5) |
| Can you please explain why you ranked the above programs in that order? | OPEN ENDED |
| Do any other suggested formats come to mind that were not listed above? | OPEN ENDED |

| Question | Response |
|---|---|
| Here are some barriers that might stop you from using brain health programs. Please rate how much you agree or disagree with these statements | I do not have time (1)<br>I do not want to spend too much money on brain health programs. (2)<br>I want an easily accessible brain health program (3)<br>I want to ensure I am educated on what to do with a brain health program (4)<br>I want to ensure I am educated on the brain health program and the outcomes of it (5)<br>If no one is holding me accountable, I will not implement brain health programs in my home (6)<br>Brain health is not something I worry about/want my child to be educated on (7)<br><br>RESPONES:<br>Strongly agree (1)<br>Agree (2)<br>Somewhat agree (3)<br>Neither agree nor disagree (4)<br>Somewhat disagree (5)<br>Disagree (6)<br>N/A (7) |
| Are there any barriers you feel may impact the implementation of positive lifestyle changes (healthy eating, physical activity, sleep schedule) in the home? | OPEN ENDED |
| What are likely to be the main difficulties or problems that stop you/your child from engaging in these programs in the long-term? | OPEN ENDED |
| Do you have any drivers or motivators that make you passionate about implementing healthy lifestyle changes in the home for your children? | Yes (1)<br>No (2) |
| What makes you passionate about healthy lifestyle habits for your child(ren)? | OPEN ENDED |
| What makes you unmotivated about healthy lifestyle habits for your child(ren)? | OPEN ENDED |
